# Reproductive plasticity in response to the changing cluster size during the breeding period: a case study in a spider mite

**DOI:** 10.1007/s10493-023-00834-y

**Published:** 2023-08-31

**Authors:** Nuwan Weerawansha, Qiao Wang, Xiong Zhao He

**Affiliations:** 1 School of Agriculture and Environment, Massey University, Private Bag 11222, Palmerston North, New Zealand; 2 Faculty of Animal Science and Export Agriculture, Uva Wellassa University of Sri Lanka, Passara Road, Badulla, 90000 Sri Lanka

**Keywords:** Dynamic social environments, Fecundity, Egg size, Fertilization, Sex allocation

## Abstract

Animals living in clusters should adjust their reproductive strategies to adapt to the social environment. Theories predict that the benefits of cluster living would outweigh the costs of competition. Yet, it is largely unknown how animals optimize their reproductive fitness in response to the changing social environment during their breeding period. We used *Tetranychus ludeni* Zacher, a haplodiploid spider mite, to investigate how the ovipositing females modified their life-history traits in response to the change of cluster size (i.e., aggregation and dispersal) with a consistent population density (1 ♀/cm^2^). We demonstrate that (1) after females were shifted from a large cluster (16 ♀♀) to small ones (1 ♀, 5 and 10 ♀♀), they laid fewer and larger eggs with a higher female-biased sex ratio; (2) after females were shifted from small clusters to a large one, they laid fewer and smaller eggs, also with a higher female-biased sex ratio, and (3) increasing egg size significantly increased offspring sex ratio (% daughters), but did not increase immature survival. The results suggest that (1) females fertilize more larger eggs laid in a small population but lower the fertilization threshold and fertilize smaller eggs in a larger population, and (2) the reproductive adjustments in terms of egg number and size may contribute more to minimize the mate competition among sons but not to increase the number of inhabitants in the next generation. The current study provides evidence that spider mites can manipulate their reproductive output and adjust offspring sex ratio in response to dynamic social environments.

## Introduction

Animals are predicted to live in clusters when the benefits of cluster living outweigh the costs (Alexander 1974; Bilde et al. 2007; Guindre-Parker et al. 2020; Tinsley Johnson et al. 2021). In cluster-living species, cluster size is an important component of social environment that influences individuals’ behaviour (Prokopy and Duan 1998; Majolo et al. 2008; Krams et al. 2009; Le Goff et al. 2010; Fryxell and Berdahl 2018) and physiology (Lihoreau and Rivault 2008; Clotuche et al. 2014; Markham et al. 2015; Markham and Gesquiere 2017; Rudolph et al. 2019), altering their life-history traits (Prokopy and Reynolds 1998; Bilde et al. 2007; Borries et al. 2008; Le Goff et al. 2010; Chapman and Valenta 2015; Vanthournout et al. 2016; Tinsley Johnson et al. 2021). Some fitness components are expected to increase with increasing cluster size, for example, the reduction of predation risk (Spieler 2003; Morrell and James 2008; Yano 2012; Saito and Zhang 2017) and increase in reproductive success (Snead and Alcock 1985; Prokopy and Reynolds 1998; Le Goff et al. 2010; Pérez-González et al. 2010; Bonsignore and Jones 2014). However, living in a large cluster may also reduce fitness because of the interference and food competition among group members (Bilde et al. 2007; Estevez et al. 2007; Grove 2012; Wong et al. 2013; Li and Zhang 2021; Tinsley Johnson et al. 2021).

In many empirical studies, researchers usually compare the effect of cluster size on fitness by maintaining individuals in consistently small or large clusters during the entire reproductive period (Avileś and Tufinõ 1998; Bilde et al. 2007; Le Goff et al. 2010; Li and Zhang 2021). However, cluster size often changes while individuals are reproducing due to frequent deaths, births, aggregation (immigration) and dispersal (emigration) (Roeder 1992; Roff 1992; Stearns 1992; Price and Hunter 1995; Bowman et al. 2002; Schausberger et al. 2021). As a result, animals must have developed reproductive plasticity to adapt to dynamic social environment to maximize the fitness of their offspring and their own (Ross et al. 2013; Radwan et al. 2014; De Roissart 2016; Weerawansha et al. 2020, 2022a, b; Tinsley Johnson et al. 2021). To date, it is still unknown how changes of cluster size during female reproductive phase affect their reproductive strategies.

Spider mites (Acari: Tetranychidae), such as invasive pests *Tetranychus ludeni* Zacher and *Tetranychus urticae* Koch, have been used as model species to examine changes of reproductive strategies mediated by social environment (Le Goff et al. 2010; Macke et al. 2012a; Weerawansha et al. 2020, 2022a, b, c, d). They live in patchy clusters of varying size and density and produce silk webs for dispersal and protection against predation and environmental hazards (Le Goff et al. 2010; Yano 2012; Schausberger et al. 2021). Furthermore, cluster-living females elevate egg production compared to solitary ones (Le Goff et al. 2010; Weerawansha et al. 2022a). However, when the cluster size becomes too large (overcrowding), the costs of competition among individuals outweigh the benefits of aggregation and females may leave the oviposition site in search of favourable habitats (Azandémè-Hounmalon et al. 2014; Li and Zhang 2021; Schausberger et al. 2021; Zhou et al. 2021), reducing the local cluster size.

Like other haplodiploid species, mated spider mite females produce haploid sons and diploid daughters (Young et al. 1986; Macke et al. 2011a; Zhou et al. 2018). They can manipulate offspring sex ratio in response to the social environment by adjusting egg size and fertilizing larger eggs that develop into daughters (Macke et al. 2011a, 2012a). Weerawansha et al. (2022a, d) report that when *T. ludeni* females are maintained in clusters of constant size during their lifetime, they lay fewer eggs but produce higher female-biased offspring in small clusters compared to large ones. This may be an adaptive strategy to minimise the local mate competition between sons (Sato and Saito 2007; Macke et al. 2012a, 2014; Weerawansha et al. 2022a, b, d). Moreover, larger eggs have a greater likelihood of developing to larger offspring that are more likely to reach maturity (Jackson and Martin 2010; Macke et al. 2011b) and more likely of higher fecundity (Zhou et al. 2018). However, whether females could optimise their fecundity and manipulate offspring sex ratio in response to the change of cluster size due to aggregation or dispersal during their breeding period is largely unknown. Such knowledge is crucial for understanding the mechanisms of population dynamics in the changing social environment and assessing the invasion success in novel habitats.

Based on the theoretical and empirical framework outlined above, we hypothesized that ovipositing females could produce larger eggs and more female-biased offspring in larger clusters than in smaller ones. Using the haplodiploid spider mite *T. ludeni*, we simulated three scenarios of cluster size changes that frequently occur in nature, i.e., dispersal (decreasing population size), aggregation (increasing population size), and residence after settling in a habitat (consistent population size), during their reproductive phase. We then measured the effects of cluster changes on female reproductive investment patterns. This study provides insight into the mechanisms of reproductive adjustments by animals for optimizing their reproductive fitness gains in response to social environmental changes during the breeding period.

## Materials and methods

### Mite colony

We started the colony of *T. ludeni* from adults collected on *Passiflora mollissima* (Kunth) in Palmerston North, New Zealand. We reared mites on kidney bean plants (*Phaseolus vulgaris* L.) and used the first expanded leaves of 1- to 2-week-old plants for the experiment. We maintained the colony and carried out the experiment in two separate environmental rooms at 25 ± 1 ºC, 40 ± 10% RH, and a L16:D8 photoperiod.

### Mite preparation

To obtain mated females, we randomly collected quiescent female deutonymphs (teleiochrysalis stages) from the colony and individually transferred them onto a 1-cm^2^ leaf square placed upside down on a wet cotton pad in a Petri dish (9.5 cm diameter × 1 cm height) with a mesh-sealed hole (1 cm diameter) in the middle of the lid. Before the female emerged (silvery in colour), we introduced a newly emerged male adult onto the leaf square. The male was produced by virgin females that developed from individually reared quiescent female deutonymphs randomly collected from the colony. The pair mated upon female emergence. The male was removed immediately after copulation ended. The newly mated females were used for the experiment. Because the mated females began to lay eggs on the emergence day and the first mortality occurred 6 days after emergence, we scheduled the shift of population size 3 days after emergence and used the data collected in the first 6 days for the analysis.

### Experimental design and data collection

To explore how cluster size changes during the breeding period of *T. ludeni* females altered their reproductive output and sex allocation, we kept the population density constant (1 ♀/cm^2^ on leaf squares in Petri dishes, as mentioned above) during the experiment and set up three scenarios: dispersal, aggregation, and residence. (1) Dispersal – we simulated female dispersal during the experimental period by transferring the ovipositing females from a large cluster of 16 individuals on a 16-cm^2^ leaf square to three small clusters of 1, 5, and 10 females on 1-, 5-, and 10-cm^2^ leaf squares, respectively, on the 4th day of oviposition. (2) Aggregation – we simulated female aggregation during the experimental period by transferring the females from three small clusters of 1, 5, and 10 females ovipositing on 1-, 5-, and 10-cm^2^ leaf squares, respectively, to a large cluster of 16 individuals on a 16-cm^2^ leaf square on the 4th day of oviposition. (3) Residence – we simulated female residence by maintaining 1, 5, 10, and 16 females on 1-, 5-, 10-, and 16-cm^2^ leaf squares, respectively, during the experimental period. Therefore, there were three treatments in scenario (1) or (2), and four treatments in scenario (3). Fifteen replicates were carried out for each treatment.

For each replicate in the ‘dispersal’ and ‘aggregation’ scenarios, we transferred females onto a new leaf square of the same size daily for three consecutive days and then shifted them to leaf squares of desired cluster size on the 4th day. The shifted females were daily transferred onto a new leaf square of the same size for the following 2 days. For each replicate in the ‘residence’ scenario, we transferred the females onto a new leaf square of the same size daily throughout the experimental period. Leaf squares were checked twice a day and any dead females were replaced immediately with those of the same age and reproductive history.

The number of eggs laid on each leaf square was recorded and the total number of eggs laid by individual females during days 1–3 and 4–6 were calculated. To reduce the workload, we measured the diameter of all, 10, 15 and 30 eggs on leaf squares with 1, 5, 10 and 16 feeding females, respectively, under a stereomicroscope (Leica MZ12, Germany) connected to a digital camera (Olympus SC30, Japan) and imaging software (CellSens GS-ST-v1.7, Olympus, Japan). We then measured the radius (*r* = diameter/2) and calculated the egg size (volume = $4/3\pi {r}^{3}$). Egg hatching on each leaf square was recorded daily, and all live individuals were transferred onto a clean leaf square of the same size once every 5 days. The number and sex of newly emerged adults were recorded. The immature survival rate was calculated as the number of emerged adults divided by the number of eggs laid.

### Statistical analysis

We analysed data using SAS v.9.4 with a reject level set at α = 0.05. Data on the egg number and size were normally distributed (Shapiro-Wilk test; UNIVARIATE procedure). Thus, for the dispersal or aggregation scenario, a linear mixed model (MIXED procedure) was applied to compare egg number and size with cluster size as a fixed factor and replicate as a random factor, and a Tukey-Kramer test for multiple least squares mean comparisons. For each residence treatment, we applied a paired t-test to compare the difference in the egg number or egg size between early (days 1–3) and late (days 4–6) reproductive episodes. The above linear mixed model was also used to compare the number of eggs laid by females after dispersal from a large cluster to small ones or aggregation from small clusters to a large one with the number of eggs laid by females in constant cluster size during their late reproductive episode. A generalized linear regression model (GLMMIX procedure) was used to analyse the data on offspring sex ratio (% daughters) and immature survival rate with a binomial distribution and a *Logit* link function after the model, and a Tukey-Kramer test was applied for least squares means multiple comparisons. A generalized linear regression model (GLMMIX procedure) with a gamma distribution and a *Log* link function was applied to determine the relationships between number and size of eggs, between egg size and sex ratio, and between egg size and immature survival rate. The mean data for each female were calculated, and data of different treatments were pooled for regressions.

## Results

### Effect of cluster size changes on fecundity

**Fig. 1:**
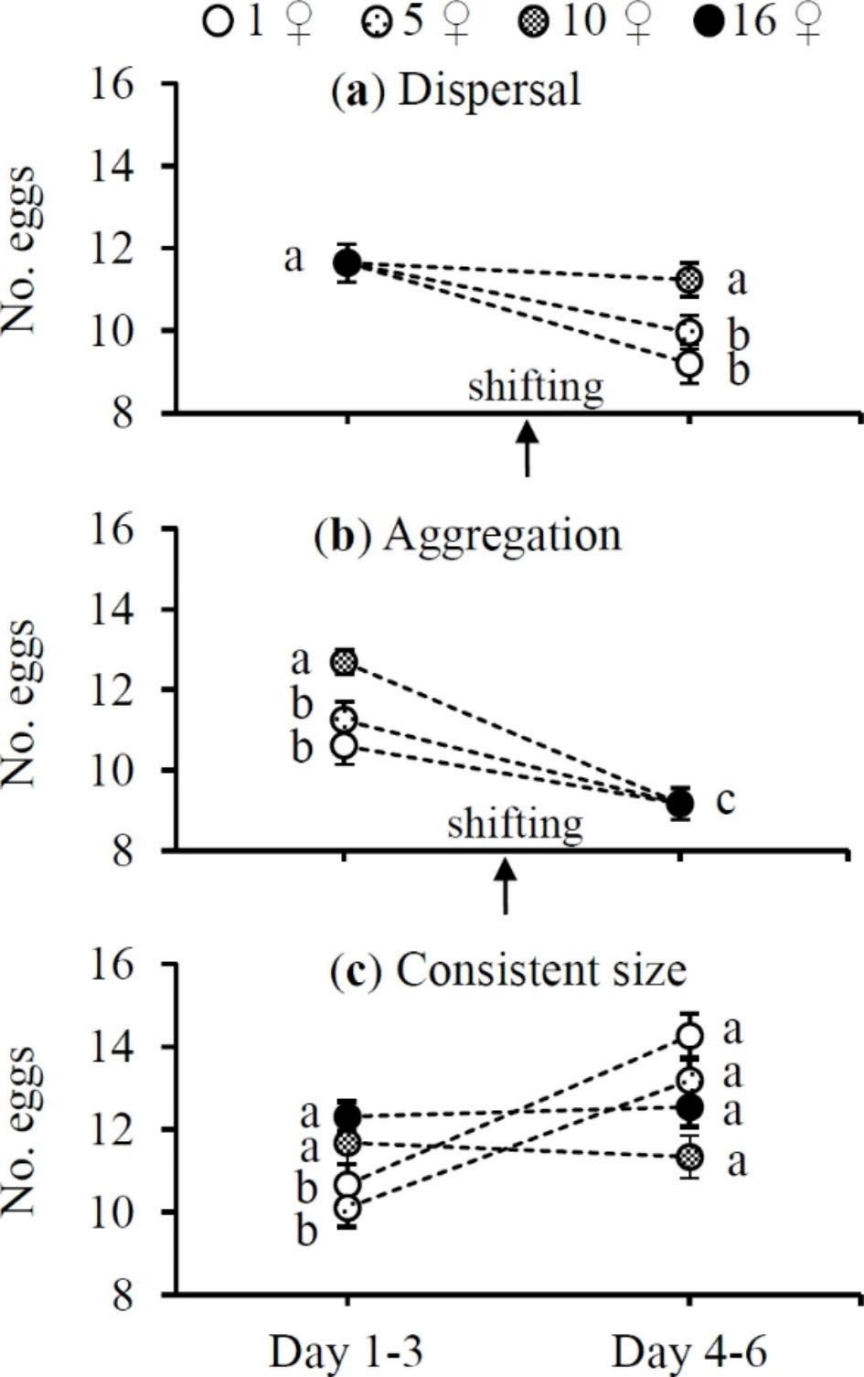
Mean (± SE) number of eggs laid by *Tetranychus ludeni* females during the early (days 1–3) and late reproductive episodes (days 4–6) when they dispersed from a large cluster to smaller ones (**a**), aggregated from smaller clusters to a larger one (**b**), or resided in clusters of consistent size (**c**). The shift in cluster size under dispersal and aggregation was performed on the 4th day of oviposition. Means within a treatment and within a panel marked with the same letter are not significantly different (paired t-tests: *P* > 0.05)

Females laid significantly more eggs in the cluster of 16 ♀♀ before transfer to smaller clusters of 1 ♀ and 5 ♀♀, where they laid fewer eggs (*F*_3,42_ = 17.86, *P* < 0.0001) (Fig. 1a). Before transfer to the cluster of 16 ♀♀, females laid significantly more eggs in the cluster of 10 ♀♀ than in clusters of 1 ♀ and 5 ♀♀, but after transfer to the cluster of 16 ♀♀, they laid fewer eggs (*F*_3,42_ = 19.80, *P* < 0.0001) (Fig. 1b). In treatments of consistent cluster size, females in clusters of 1 and 5 ♀♀ laid more eggs during their late reproductive episode compared to the early one (*t*_14_ = -2.82 and -5.28 for 1 ♀ and 5 ♀♀, respectively; both *P* < 0.05) (Fig. 1c), whereas females in clusters of 10 and 16 ♀♀ had similar fecundity in both reproductive episodes (*t*_14_ = 0.43 and -0.76 for 10 and 16 ♀♀, respectively; both *P* > 0.05) (Fig. 1c).

**Fig. 2:**
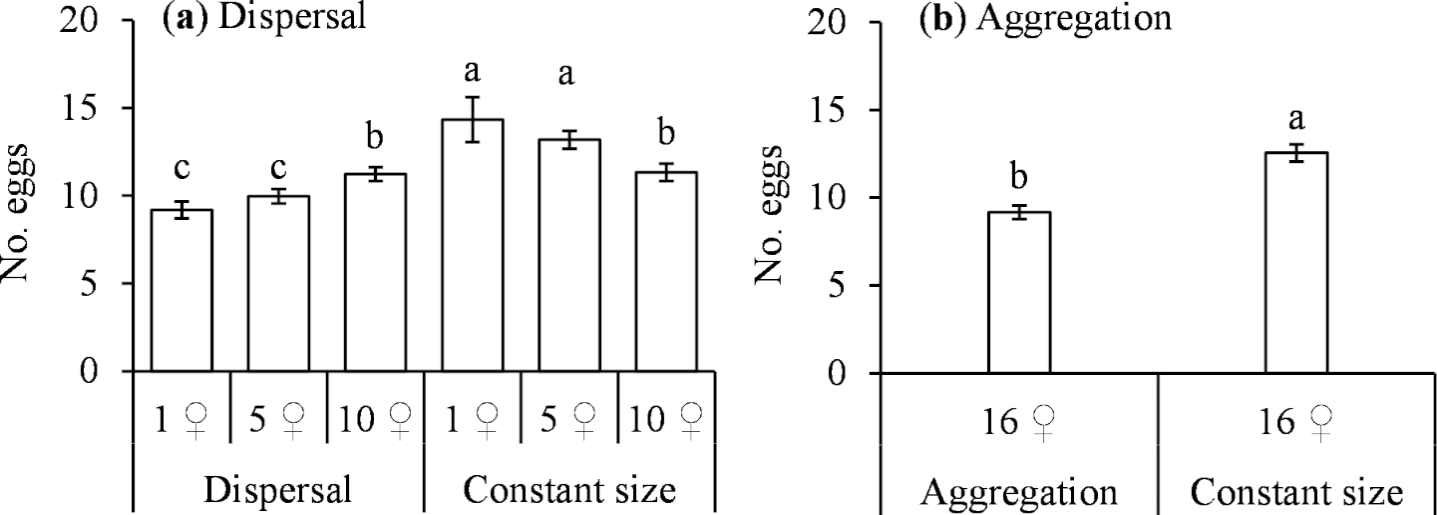
Comparison of mean (± SE) number of eggs laid by *Tetranychus ludeni* females during their late reproductive episode (days 4–6) in scenarios of dispersal (**a**) and aggregation (**b**). Means within a panel capped with the same letter are not significantly different (Tukey-Kramer test: *P* > 0.05)

The numbers of eggs laid by *T. ludeni* females after dispersal from a large cluster to small ones or after aggregation from small clusters to a large one were lower than the number of eggs laid by females in a constant cluster size during their late reproductive episode (dispersal: *F*_5,70_ = 11.82; aggregation: *F*_1,14_ = 77.11, both *P* < 0.0001) (Fig. 2). However, females that dispersed from 16 to 10 ♀♀ and females that resided in constant cluster of 10 ♀♀ laid similar numbers of eggs during their late reproductive episode (P > 0.05) (Fig. 2a).

### Effect of cluster size changes on egg size

**Fig. 3:**
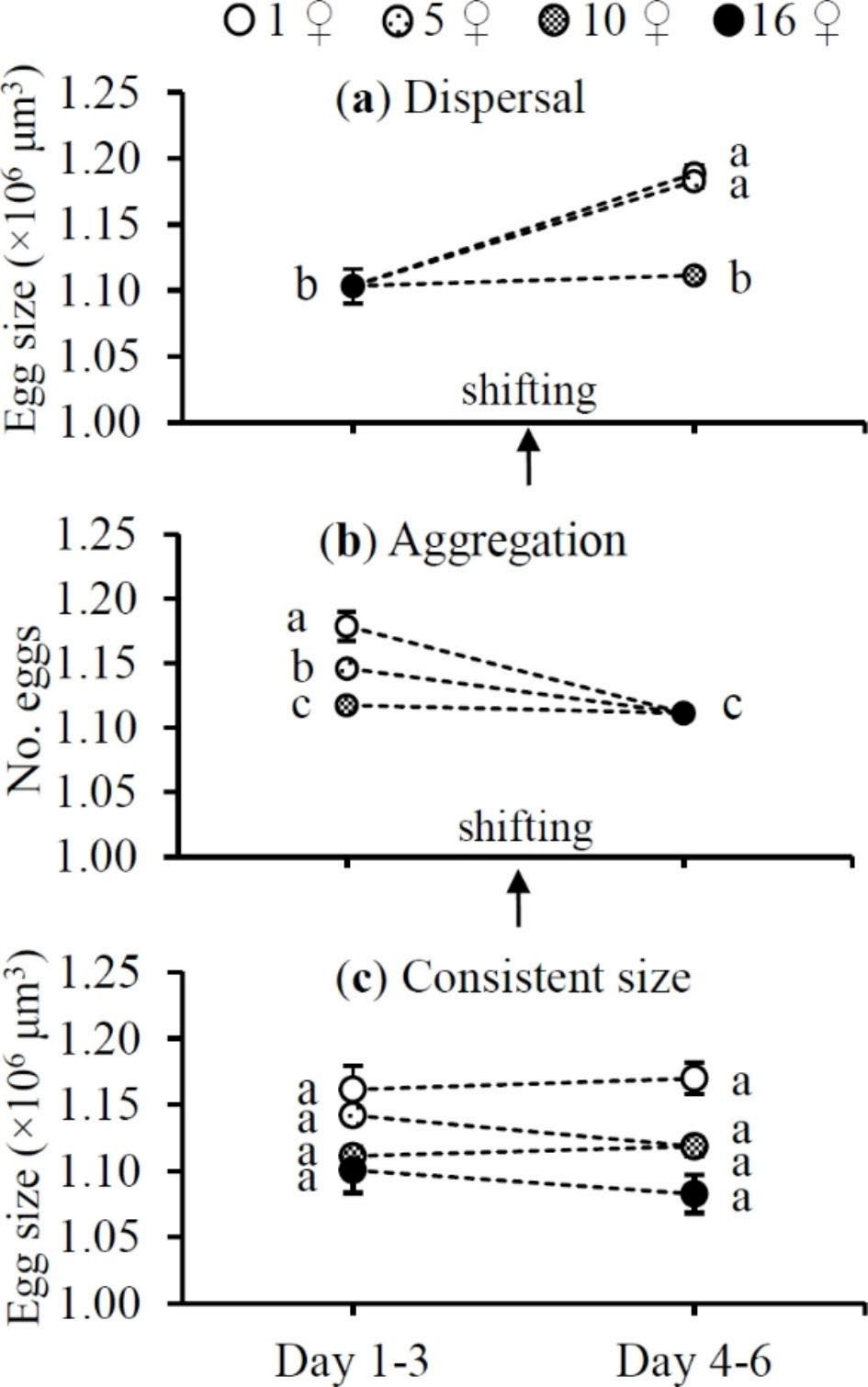
Mean (± SE) size of eggs laid by *Tetranychus ludeni* females during the early (days 1–3) and late reproductive episodes (days 4–6) when they dispersed from a large cluster to smaller ones (**a**), aggregated from clusters groups to a large one (**b**), or resided in clusters of consistent size (**c**). The shift in cluster size under dispersal and aggregation was performed on the 4th day of oviposition. Means within a treatment and within a panel marked with the same letter are not significantly different (Tukey-Kramer test: *P* > 0.05)

**Fig. 4:**
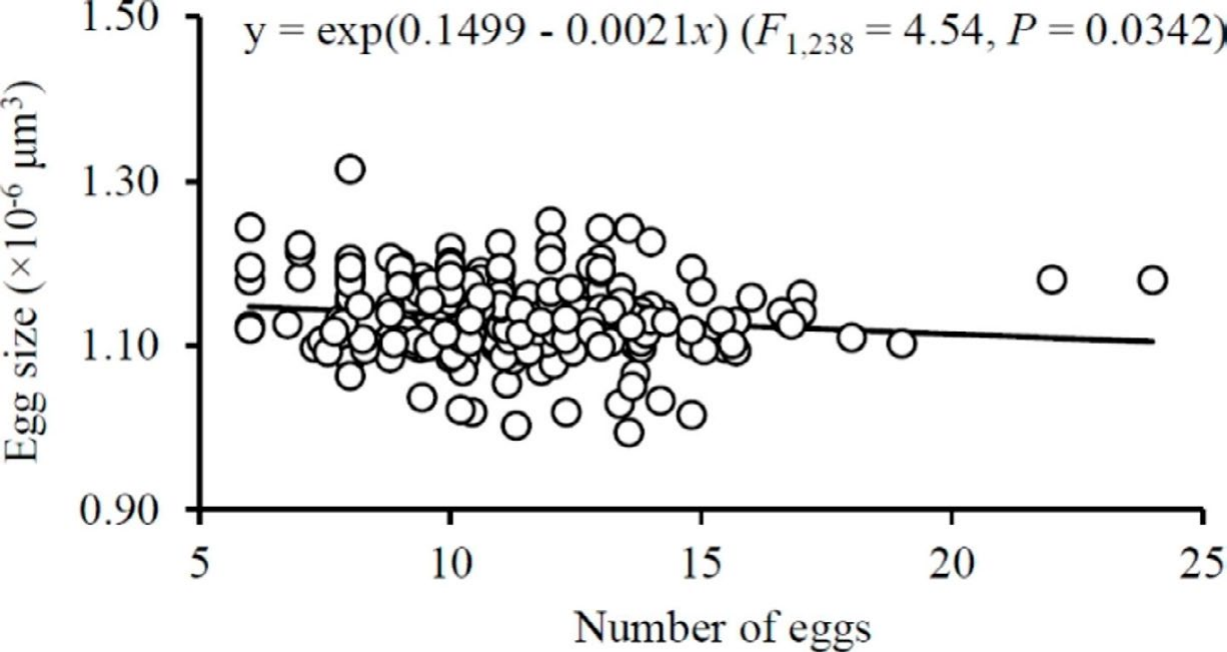
Relationship between egg size and egg number in *Tetranychus ludeni*

Females laid larger eggs after shift from the cluster of 16 ♀♀ to clusters of 1 ♀ and 5 ♀♀ but smaller eggs after transfer from clusters of 1 ♀ and 5 ♀♀ to the cluster of 16 ♀♀ (*F*_3,42_ = 122.31 and 95.19 for dispersal and aggregation scenarios, respectively; both *P* < 0.0001) (Fig. 3a–b). If cluster size remained consistent, egg size did not change during the two reproductive episodes (*t*_14_ = -0.43, 2.10, -2.13 and 1.44 for treatments of 1 ♀, 5, 10 and 16 ♀♀, respectively; all *P* > 0.05) (Fig. 3c). Furthermore, egg size significantly decreased with the increase of egg numbers (Fig. 4).

### Effect of cluster size changes on sex ratio and survival of offspring

**Fig. 5:**
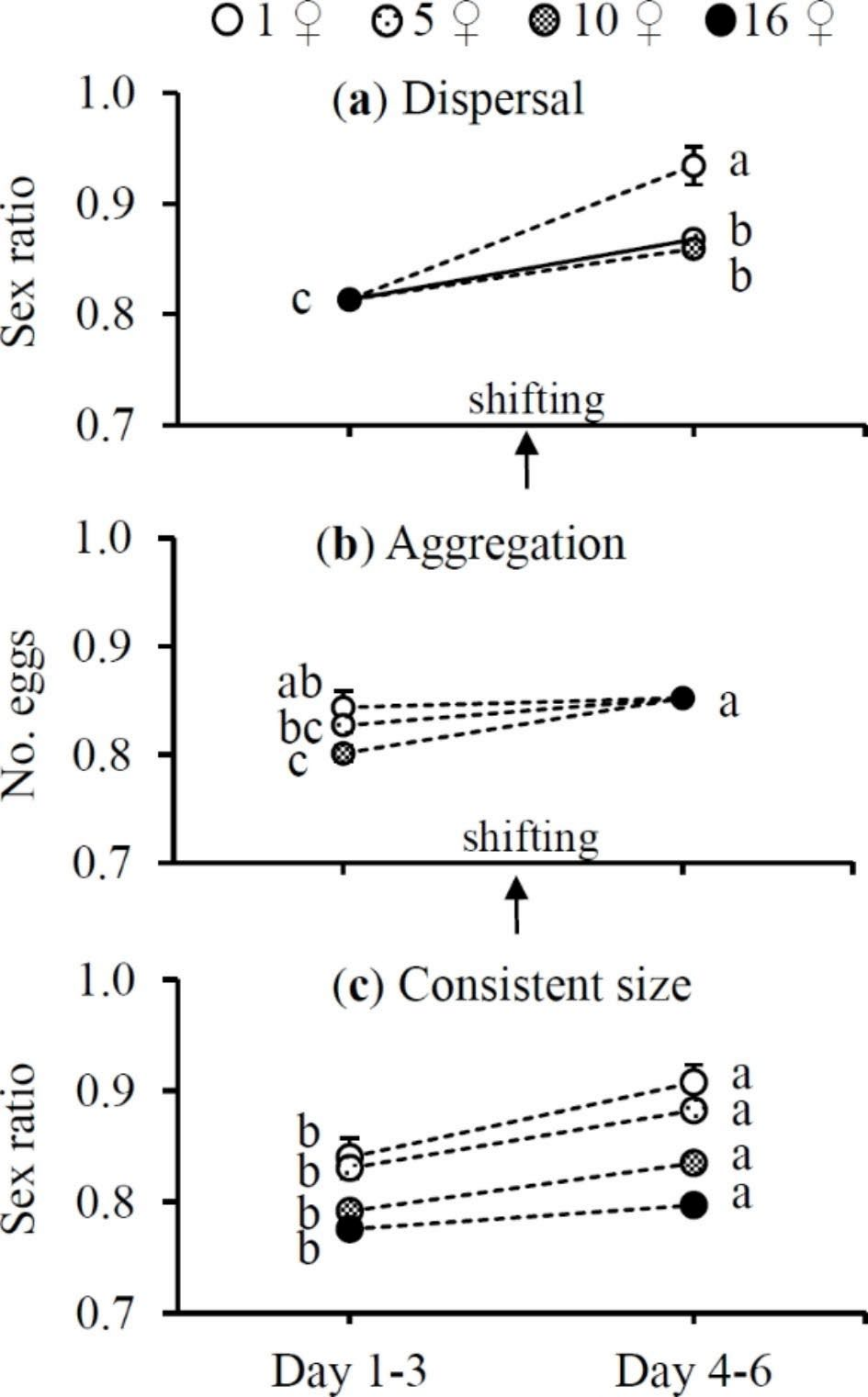
Mean (± SE) sex ratio (% daughters) of *Tetranychus ludeni* females during the early (day 1–3) and late reproductive episodes (day 4–6) when they dispersed from a large cluster to smaller ones (**a**), aggregated from smaller clusters to a large one (**b**), or resided in clusters of consistent size (**c**). The shift in cluster size under dispersal and aggregation was performed on the 4th day of oviposition. Means within a treatment and within a panel marked with the same letter are not significantly different (Tukey-Kramer test: *P* > 0.05)

Offspring sex ratio was female-biased (77.5–93.5% daughters) regardless of cluster size where mothers lived (Fig. 5). After transfer from large to small clusters, females produced more female-biased offspring (*F*_3,42_ = 8.93, *P* < 0.0001) (Fig. 5a) but after transfer from clusters of 5 and 10 ♀♀ to the cluster of 16 ♀♀, they also generated more female-biased offspring (*F*_3,42_ = 5.80, *P* = 0.0021) (Fig. 5b). Regardless of the shifting scenarios, the sex ratio (% daughters) was significantly higher in the cluster of 1 ♀ than in the cluster of 10 ♀♀ (Fig. 5a–b). If cluster size remained consistent, females produced more female-biased offspring in the late reproductive episode than in the early one (*F*_1,27_ = 4.39, 7.07, 8.02 and 4.23 for 1 ♀, 5, 10 and 16 ♀♀, respectively; all *P* < 0.05) (Fig. 5c).

**Fig. 6:**
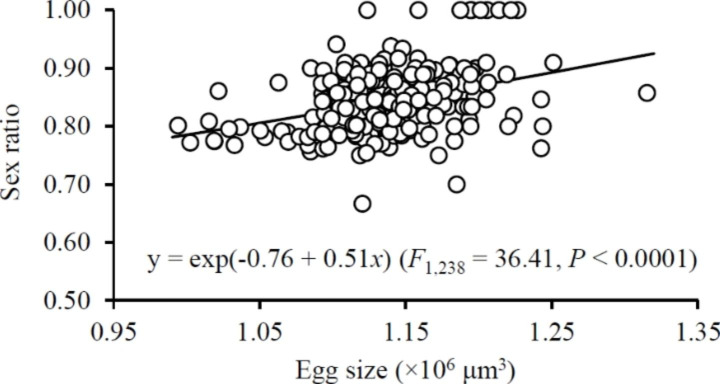
Relationship between sex ratio (% daughters) and egg size in *Tetranychus ludeni*

**Fig. 7:**
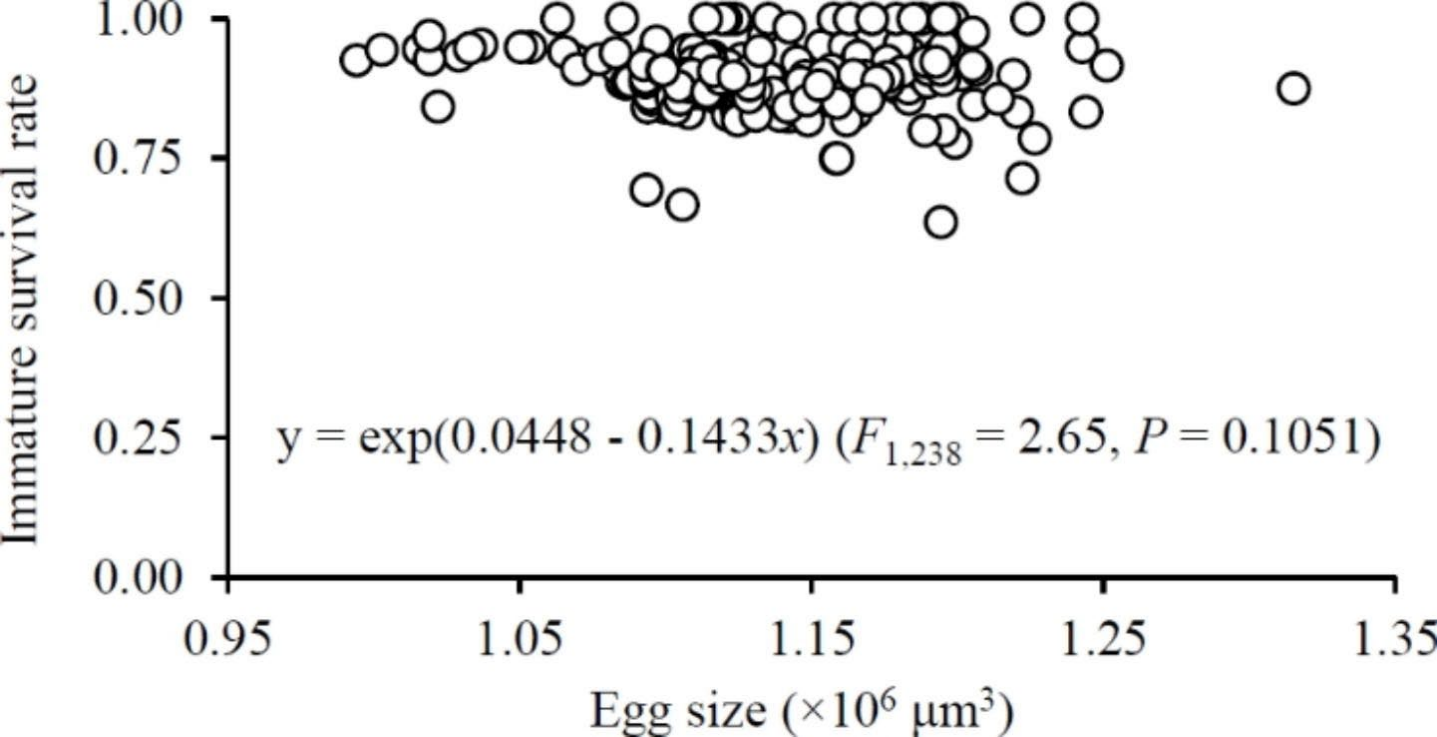
Immature survival rate in relation to egg size in *Tetranychus ludeni*

A significant positive relationship was detected between sex ratio and egg size (Fig. 6). The mean immature survival rate ranged between 0.85 and 0.94 which was not different between the early and late reproductive episodes regardless of the shifting scenarios (*F*_3,42_ = 2.08 and 1.95 for dispersal and aggregation scenarios, respectively; both *P* > 0.05) or for clusters of consistent size (*F*_1,27_ = 1.76, 0.03, 2.73 and 0.61 for 1 ♀, 5, 10 and 16 ♀♀, respectively; all *P* > 0.05). Egg size had no significant impact on immature survival rate (Fig. 7).

## Discussion

We demonstrate that *T. ludeni* females could adjust their reproductive strategies in response to changes in cluster size during the breeding period. As expected, the ovipositing females laid significantly more eggs in a large cluster of 16 ♀♀ before they dispersed and settled in smaller clusters of 1 ♀, 5, and 10 ♀♀ (Fig. 1a). It is well known that spider mites collectively spin common silk webs, which help disperse and protect them from predation and environmental hazards (Le Goff et al. 2010; Yano 2012; Schausberger et al. 2021). Nevertheless, silk is composed mainly of proteins, and thus its production costs energy and nutrient reserved (Hazan et al. 1975; Oku et al. 2009). Living in a cluster may confer the advantage of sharing the spun webs or reducing the intensity (i.e., thinner, shorter, and/or fewer silks) of web production (Hazan et al. 1975) so that the energy and nutrient saved in web production can be invested in reproduction (Oku et al. 2009; Le Goff et al. 2010). In addition, cluster-living individuals could benefit from modifying plant biochemistry, such as breaking down the plant defence system, resulting in more favourable nutritional quality of the shared host plants (Kant et al. 2008; Rioja et al. 2017), which may also elevate female reproduction.

We further find that females in small clusters of consistent size (i.e., 1 ♀ and 5 ♀♀) laid significantly more eggs in the late reproductive episode than in the early one, but those in large clusters of consistent size (i.e., 10 and 16 ♀♀) had similar fecundity during the two reproductive episodes (Fig. 1c). Following this line, it is expected that in the dispersal and aggregation scenarios, females would increase or at least maintain the fecundity in late reproduction. However, contrary to expectation, females reduced reproduction after they were shifted from a large to small clusters (i.e., 1 ♀ and 5 ♀♀) (Figs. 1a and 2a) or from small clusters to a large one (Figs. 1b and 2b). We suggest that ovipositing females might constrain their reproduction in response to cluster size changes. Previous experimental and theoretical studies have also shown that a lower reproductive rate in response to unpredictable environments can confer a long-term selective advantage about population persistence (Gilpin 1975; Nathanson 1975; Wade 1980; Sober and Wilson 1998; Reed et al. 2010).

In the present study, we show a consistent pattern of adjustment in egg size and offspring sex ratio of *T. ludeni* females in smaller clusters, i.e., 1 ♀ > 5 ♀♀ > 10 ♀♀, regardless of their shifting scenarios (Figs. 3a-b and 5a-b) and demonstrate a significantly positive relationship between egg size and offspring sex ratio (Fig. 6). These results suggest that *T. ludeni* females could promote the offspring fitness by producing larger eggs and more daughters, and thus reducing the intensity of mate competition between their sons in smaller clusters (Macke et al. 2011a, 2012a; Weerawansha et al. 2022a, b, d). We further reveal that after aggregating into a large cluster, females laid significantly smaller eggs (Fig. 3b) but produced a significantly higher female-biased sex ratio (Fig. 5b), which may be attributed to the flexibility of egg fertilization in spider mites. Macke et al. (2011b) report that in spider mites, mated females will fertilize eggs when the size of those eggs exceeds a threshold value. Therefore, our results suggest that *T. ludeni* females could adjust the fertilization threshold to a lower level and fertilise relatively smaller eggs.

In consideration of the fact that mothers have finite resources partitioned to their offspring (Bernardo 1996; Fox and Czesak 2000), they can either produce many small or a few large offspring with a balance or trade-off between egg number and size (Roff 1992; Einum and Fleming 2000; Krist 2011; Morrongiello et al. 2012). In spider mites, as females allocate more resources to fertilised eggs (Macke et al. 2012a, 2012b), it is not surprising that increasing egg size induced a significant decrease of egg number in this study (Fig. 4). However, increasing egg size had no significant impact on immature survival (Fig. 7), suggesting that females increasing egg provision may contribute to the female-biased offspring sex ratio (Fig. 6) rather than the survival of offspring (Fig. 7). Therefore, in response to the variations of cluster size, the resource allocation in terms of egg number and size will minimise the mate competition among sons (i.e., high female-biased offspring sex ratio), rather than increase the number of inhabitants in the next generation.

In summary, we show that *T. ludeni* females lay more eggs in a large cluster before they are shifted to the small ones probably due to the enhanced cooperation in spinning webs so that the conserved energy and resources could be invested in reproduction. We first demonstrate that the ovipositing females constrain reproduction in response to the alternation of social environment during their reproductive period. We further reveal that females trade off egg number for size and fertilise larger eggs that are more likely to give rise to daughters, and they could lower the fertilization threshold to fertilise more eggs when egg size is small in response to the alternation of social environment. However, increasing egg size does not increase immature survival, thus females increasing provisioning in egg size is to promote the female-biased sex ratio rather than the survival of offspring. This study provides insight into the adaptive responses of haplodiploid animals to varying social environments.
